# Voluntary Smoke-Free Home Rules and Exposure to Secondhand Smoke in Poland: A National Cross-Sectional Survey

**DOI:** 10.3390/ijerph17207502

**Published:** 2020-10-15

**Authors:** Mateusz Jankowski, Jarosław Pinkas, Wojciech S. Zgliczyński, Dorota Kaleta, Waldemar Wierzba, Mariusz Gujski, Vaughan W. Rees

**Affiliations:** 1 School of Public Health, Centre of Postgraduate Medical Education, 01-826 Warsaw, Poland; mjankowski@cmkp.edu.pl (M.J.); jpinkas@cmkp.edu.pl (J.P.); wzgliczynski2@cmkp.edu.pl (W.S.Z.); 2 Center for Global Tobacco Control, Department of Social and Behavioral Sciences, Harvard T.H. Chan School of Public Health, Boston, MA 02115, USA; 3 Department of Hygiene and Epidemiology, Medical University of Lodz, 90-647 Łódź, Poland; dorota.kaleta@umed.lodz.pl; 4 UHE Satellite Campus in Warsaw, University of Humanities and Economics in Łódź, 01-513 Warsaw, Poland; wwierzba@post.pl; 5 Department of the Prevention of Environmental Hazards and Allergology, Medical University of Warsaw, 02-091 Warsaw, Poland; mariusz.gujski@wum.edu.pl

**Keywords:** secondhand smoke, smoke-free policy, exposure, tobacco control, prevalence, smoke-free home rule, smoking ban

## Abstract

Smoke-free policies have been shown to significantly reduce secondhand smoke (SHS) exposure in private and public places. The objectives of this study were to: (1) to assess the prevalence and characteristics of voluntary smoke-free home rules in Poland; and (2) assess the association of smoke-free rules with self-reported SHS exposure in private homes. A cross-sectional survey was conducted in September 2019 with a nationally representative sample of 1011 individuals aged 15 and over. Nationally, 66.1% of individuals had a 100% smoke-free home rule (78.9% of non-smokers and 18.6% of smokers; *p* < 0.001), while a further 24.6% had adopted a partial home smoking rule. SHS exposure in the home during past month was reported by 6.1% of respondents (11.5% of smokers and 4.5% of non-smokers; *p* < 0.001). The lowest level of SHS exposure (1.8%) was observed among respondents who had implemented a full smoke-free home rule. Non-smokers had higher odds of having adopted a total smoke-free home rule compared with smokers (aOR: 19.17; 95% CI: 12.89–28.50). Moreover, non-smokers had lower odds (aOR: 0.35; 95% CI: 0.20–0.61; *p* < 0.001) of self-reporting SHS smoke exposure at home. Although two-thirds of the Polish population have adopted a full smoke-free home rule in their homes, smokers continue to lag in adoption rates relative to non-smokers.

## 1. Introduction

Secondhand smoke (SHS) is a mixture of smoke generated from burning tobacco products (e.g., cigarettes) and smoke that has been exhaled or breathed out by the smoker [1]. According to the World Health Organization (WHO), SHS exposure contributes to the premature death of approximately 1.2 million people globally each year [2]. Most exposure to SHS occurs in workplaces, homes and cars [3,4]. Because there is no safe level of exposure to SHS, public health authorities, including the U.S. Surgeon General [5], the European Commissioner for Health and Consumer Policy [6] and International Agency for Research on Cancer [7] have advised that protection is achieved most effectively with comprehensive (i.e., 100%) smoke-free policies.

In line with Article 8 of the WHO Framework Convention on Tobacco Control (WHO FCTC), many countries have adopted smoke-free laws designed to provide protection from SHS exposure in workplace and public venues [8]. However, while smoke-free policies have been shown to significantly reduce SHS exposure in work and public places, many children and non-smokers continue to be exposed to SHS in private setting (including homes) where implementation of smoke-free rules is largely voluntary [9,10].

Voluntary smoke-free home rules not only protect non-smokers from SHS exposure, but can also drive changes in smoking behavior [11,12]. The adoption of smoke-free rules in private settings is associated with increased cessation attempts and lower cigarette consumption [11,13,14]. Smoke-free homes may also help to prevent relapse among those who have quit smoking [14]. Moreover, adoption of a 100% smoke-free home rule may decrease the likelihood of smoking initiation among adolescents [15].

The prevalence of complete smoke-free home rules varies by country and global region, ranging from 6.4% in Indonesia [16], 7.6% in Pakistan [16], 55.1% in Japan [17], and up to 83.7% in the U.S. [18]. The Global Adult Tobacco Survey (GATS; 2008–2011) also reveals substantial variation in home-based SHS exposure, ranging from 17.4% in Mexico to 73.1% in Vietnam [19]. Data from the last GATS in Poland (2009–2010) found that only 37.4% of Poles (50.2% among nonsmokers and 15.7% among smokers) were protected by a full smoke-free home rule [20]. Further, 44.2% of Poles reported exposure to SHS at home in the last 30 days (28% of nonsmokers and 80.9% of smokers) [21].

Poland has made substantial progress in tobacco control in the past decade, with the adoption of smoking bans in public places, restrictions on advertising and marketing, text health warnings and other broad-based actions in 2010 [22]. Between 2011 and 2019 the prevalence of adult daily smoking (aged 15 and over) decreased from 31% to 21.0% [23]. However, it is not known whether the broad-based policy interventions of 2010 and the resulting changes in tobacco use trends have influenced the voluntary adoption of smoke-free rules in private homes. Previous studies suggest that legislative bans on smoking in public venues may also increase the prevalence of homes with a voluntary smoke-free policy [24,25]. Likewise, smoke-free home rules are more likely to be observed in jurisdictions with lower smoking prevalence [10,16].

Therefore, the objectives of this study were to: (1) to assess the prevalence and characteristics of voluntary smoke-free home rules in Poland; and (2) assess the association of smoke-free rules with self-reported SHS exposure in private homes.

## 2. Materials and Methods

### 2.1. Study Design and Population

Data for this study were provided by the Chief Sanitary Inspectorate: a Polish government agency responsible for regular monitoring of tobacco use and prevention policies. A cross-sectional survey was conducted in September 2019 with a nationally representative sample of 1011 individuals aged 15 and over [23]. Data were collected by a specialized survey company using a computer-assisted personal interviewing (CAPI) technique, on behalf of the Chief Sanitary Inspectorate [26]. A random quota sample was selected with an address-based sampling frame [27]. The stratification model was based on demographic data from the national population report and includes gender, age, as well as the size of domicile and the territorial distribution within administrative units.

Participation in the study was voluntary and anonymous. The study protocol was approved by the Ethical Review Board at the Centre of Postgraduate Medical Education, Warsaw, Poland (consent number 51/PB/2020).

### 2.2. Measures

Smoking status: Respondents were asked about their smoking status, using the questions: “Have you smoked at least 100 cigarettes (or similar amount of other tobacco products) in your lifetime?” and “Do you currently smoke?” Smokers were respondents who reported having smoked at least 100 cigarettes (or other combusted tobacco products) during their lifetime and who reported current smoking (last 30 days). Non-smokers were respondents who reported having smoked fewer than 100 cigarettes during their lifetime and/or do not currently smoke.

Smoke-free rules in home: Respondents were asked about smoke-free rules in their home, using the question: “Is tobacco smoked in your home?” with four possible answers: “Yes, without limitations, i.e., throughout the house” or “Yes, but only in designated, closed areas” or “Yes, but only outside, e.g., on the balcony or terrace” or “No, my home is smoke-free (total ban on smoking)”. Based on the response to this question, smoke-free rules were categorized as: full smoke-free home rule (i.e., full ban), partial smoke-free home rule (smoking in designated closed areas and smoking on the balcony or terrace), and no smoke-free home rule.

Secondhand smoke exposure: Exposure to SHS at home in the past month was assessed using the question: “In the last month, were you exposed to second-hand smoke in your home (that is, anyone smoked inside while you were present)?”.

Sociodemographics: Questions related to sociodemographic data included: gender, age, marital status, occupational status, educational level, income class, number of household members, number of children living in the home and place of residence. Active occupational status included employees and self-employed; passive occupational status included the unemployed, students or pensioners/retirees (people who have temporarily or permanently left the workforce owing to age or disability). Household income was assessed with the question: “How do you assess your own/your family’s financial situation? (High/Medium/Low)”. Place of residence was defined by the official administrative definition of rural and urban (residence in a city regardless of the number of inhabitants).

### 2.3. Statistical Analysis

The distribution of categorical variables was shown by weighted frequencies and proportions with 95% confidence intervals (CI). Categorical variables were compared using the independent samples χ^2^ test. The univariable and multiple logistic regression analyses were conducted to calculate the odds ratios (ORs) and 95% confidence interval (CI) of selected variables in relation to the (1) implementation of a full smoke-free home rule in home and (2) exposure to SHS at home. Statistical inference was based on the criterion *p* < 0.05.

Associations between smoking status, personal characteristics (gender, age), educational level and housing characteristics (number of household members, having children in home) with implementation of a complete voluntary smoke-free home rule were conducted using logistic regression analyses. Model 1 includes demographic covariates (including gender and age) to assess their association with smoke-free home rule adoption. Model 2 adds educational level (a proxy for socio-economic position). Model 3 adds domestic factors (number of household members and number of children at home).

Additional linear regression models were constructed for the outcome of SHS exposure at home by selected socio-economic factors. Model 1 uses an ordinary least assess the effect of sex, age and smoking status on exposure to SHS at home. Model 2 adds educational level. Model 3 adds selected housing variables (number of household members and number of children at home).

Data were analyzed with SPSS, version 25 (IBM, Armonk, NY, USA).

## 3. Results

Analyses were conducted on responses from n = 1011 individuals aged 15 and over (52.1% female). Table 1 shows the characteristics of the study sample classified by smoking status. There were significant differences (*p* < 0.05) between smokers and non-smokers by gender, age, marital status, educational level and number children in home. Nationally, 66.1% of individuals had 100% smoke-free home rules, 24.6% had implemented a partial smoke-free home rule and 9.3% had not implemented any smoke-free home rules. The majority of non-smokers (79.8%) had adopted a full smoke-free home rule (Table 2). Nevertheless, a full smoke-free home rule was declared only by 18.5% of smokers (*p* < 0.001). More than a third of smokers (35.0%) had no voluntary policy regarding smoking in the home.

SHS exposure in the home during past month was reported by 6.1% of respondents (11.5% of smokers and 4.5% of non-smokers; *p* < 0.001). The lowest level of SHS exposure (1.8%) was observed among respondents with a full smoke-free home rule (Table 2).

The results of the univariate and multivariate regression analyses are presented in Table 3 and Table 4. In univariate analysis, non-smokers (OR: 17.20; 95% CI: 11.79–25.07; *p* < 0.001) and those with a secondary education or higher (OR: 1.37; 95% CI: 1.05–1.78; *p* < 0.05) had greater odds of reporting a full smoke-free home rule (see Table 3). When adjusted for all covariables, only smoking status was significantly (*p* < 0.001) associated with greater odds of having a 100% smoke-free home rule (non-smokers aOR: 19.17; 95% CI: 12.89–28.50). Non-smokers had lower odds (aOR: 0.35; 95% CI: 0.20–0.61; *p* < 0.001) of secondhand smoke exposure at home, even after controlling for other factors (Table 4).

## 4. Discussion

This study revealed that 66.1% of Polish adults report having a full smoke-free home rule. This outcome represents a marked increase over the rate of 37% reported in 2010. Even so, the current proportion of Polish households with a complete home smoking ban is lower than recently reported in the U.S. (83.7%) [18], but higher compared to other countries, including Indonesia (6.4%) [16], Pakistan (7.6%) [16] or Japan (55.1%) [17]. Despite a dramatic increase in home-based protection from SHS exposure from 2010 to 2019, a substantial proportion of Poles still live in homes with limited or no protection from SHS. The lack of protection is not evenly distributed across the population: non-smokers had 19.2 times higher odds of having a 100% smoke-free home compared to smokers. One practical response to this finding would be to implement future tobacco control initiatives that emphasize the benefit of adopting a full smoke-free home rule among smokers. In addition, we found that 6.1% of Polish adults reported being exposed to SHS in the home. Even though home-based SHS exposure occurs among a relatively low proportion of Polish adults, continued efforts are required to further reduce exposure, through targeted communications highlighting the health risks of SHS and the need to adopt a total smoke-free home rule.

While the prevalence of total smoke-free home rules among non-smokers increased by 59% (from 50.2% to 79.8%) between 2010 to 2019, the prevalence among smokers increased by just 18.5% (from 15.7% to 18.6%) [28]. This finding is reflected in data on home smoking rule adoption rates across the EU [29,30]. According to the EUREST-PLUS Project, which includes six EU countries [29], the proportion of 100% smoke-free homes among smokers in 2016 varied from 13.1% in Spain to 35.5% in Hungary [29]. In our study, a full smoke-free home rule was reported by 18.6% of smokers, which is almost half the rate observed in Hungary [28]. In a study carried out between 2011 and 2015, 61% of Italians adopted a full smoke-free home rule (32% of smokers and 69% of non-smokers) [30], comparing closely to findings observed in the present study. Because data on the prevalence of complete voluntary smoke-free home rules in EU households are limited, this topic should be included in future Eurobarometer surveys.

Between 2009–2011 and 2019, a substantial proportion of households adopted a total smoke free home rule, representing remarkable progress towards protection from home-based SHS exposure [28]. The proportion of Poles exposed to SHS at home decreased seven-fold in the past decade, from 44.2% in 2009–2011 to 6.1% in 2019 [28]. Exposure to SHS at home decreased significantly among both smokers (from 80.9% to 11.5%) and non-smokers (from 28% to 4.5%) [28]. An analysis based on data from 15 countries participating in GATS revealed that home SHS exposure is higher among those of lower income and educational attainment [19]. In contrast, we found no association between socio-economic factors and the prevalence of home SHS exposure. Indeed, our findings revealed that a total home smoke-free home rule was the only consistent factor associated with lower SHS exposure. Our study was carried out in the general population and the percentage of subjects exposed to SHS at home was relatively low (n = 62). The failure to observe an association between socio-economic factors and home SHS exposure is likely the consequence of a lack of statistical power, although confounding factors in the model, particularly smoking status, may also undermine any potential association. Certainly, further research will be needed to determine whether SHS exposure is associated with low SES in Poland.

The precise reasons for accelerated adoption of smoke free home rules in Poland cannot be determined in this cross-sectional survey. Nonetheless, there are a number of factors that could impact public attitudes towards the adoption of smoke-free rules. Of particular significance are data showing that the prevalence of daily smoking among adults Poles (aged 15 and over) fell from 31% in 2011 to 21% between 2011–2019 [23]. With a lower national rate of adult smoking, it is reasonable to assume that there will be increasingly fewer households where smoking continues to be allowed indoors. Secondly, in 2010 Poland adopted new tobacco control regulations that disallowed smoking in workplaces and 12 other types of public venues, including health care, hospitality and sporting venues [22]. Elimination of smoking in public venues is perceived as a powerful tool to shape social norms that discourage tobacco use, and so may promote the voluntary adoption of smoke-free home rules [31]. This hypothesis is supported by previous studies on tobacco control carried out biannually by the Chief Sanitary Inspectorate [23,28]. According to the 2013 national cross-sectional survey on tobacco use in Poland, 55% of respondents (72% of non-smokers and 14% of smokers) had adopted a full smoke-free home rule [28]. Within three years of the adoption of Poland’s smoke-free law, the prevalence of voluntary smoke-free home rules increased by 47% (from 37.4% in 2009–2010 to 55.0% in 2013). However, in the next six years, from 2013–2019, the proportion of 100% smoke-free homes in Poland increased further by only 20.2%. One interpretation of these trends is that the changing trajectory of the adoption of smoke-free home rules was initially driven by the introduction of a ban on tobacco use in public places in 2010. The national smoke-free laws may have been a particularly potent signal of changing social acceptability of smoking in the presence of non-smokers, including in private homes.

Despite the observation of a significant increase in the proportion of households with a full smoke-free home rule, there is still progress to be made. Our findings indicate that smokers are less likely to have a full smoke-free home rule and therefore are at higher risk of SHS exposure in the home. While wide-ranging information campaigns have been conducted to inform the public about public smoke-free laws in Poland, similar campaigns to promote voluntary smoke-free home rules have not been undertaken. In addition, ongoing efforts are needed to inform the public about the health risks of SHS exposure. According to the Public Opinion Research Center (CBOS) data, 42% of Poles reported that experiencing smoking in their presence is acceptable [32]. Moreover, between 1993 and 2016, the percentage of Poles who declared a commitment to protecting their own health increased by 27 points in total [33]. Social changes in the pro-health behaviors of Poles may have an impact on the implementation of smoke-free home rules [33]. Smoke-free home rules may themselves shape social norms and discourage tobacco use. Children who are raised in smoke-free homes are less likely to start smoking in the future [10,11]. Thus, efforts to promote smoke-free homes in Poland are likely to yield substantial longer-term impact by rendering smoking less socially acceptable, thereby reducing smoking rates and lowering SHS exposure.

This study has several limitations. First, our analytic approach assumed a simple random sample rather than using techniques appropriate for complex survey design, which may elevate the likelihood of Type I error. Nonetheless, we note that the major significant outcomes involved the influence of smoking status on smoke-free rule status and secondhand smoke exposure; a likely robust effect that is consistent with published research. Second, SHS exposure was defined based on self-reported data. We cannot exclude potential misinterpretation of questions related to SHS exposure, especially among smokers, which may invoke error such as demand bias or recall bias. Third, self-reported SHS exposure was not verified by objective measures of ambient SHS (such as passive nicotine dosimetry or concentration of PM_2.5_ particulate matter) or biomarkers of SHS exposure [34]. Environmental or biochemical indices of SHS exposure may help to address problems of under- or over-reporting SHS exposure.

Despite these limitations, the data reported here represent the most comprehensive insight to date on voluntary smoke-free home rules in Poland, while highlighting the significant practical implications of tobacco control actions that may increase the adoption of smoke free home rules.

## 5. Conclusions

Although two-thirds of the Polish population have adopted a total smoke free home rule, smokers lag in their rate of adoption relative to non-smokers. The present data underscore the importance of adopting a full smoke-free home rule to protect household members from exposure to secondhand smoke.

## Figures and Tables

**Table 1:** Study sample: characteristics by smoking status.

Variable	Total N = 1011		Smokers n = 226		Non-Smokers n = 785		*p*
		Weighted		Weighted		Weighted	
	n	% (95% CI)	n	% (95% CI)	n	% (95% CI)	
**Gender**							
Men	484	47.9 (44.8–51.0)	125	55.3 (48.8–61.7)	359	45.7 (42.3–49.2)	0.01
Women	527	52.1 (49.1–55.2)	101	44.7 (38.4–51.2)	426	54.3 (50.8–57.7)	
**Age (years)**							
15–19	63	6.2 (4.9–7.9)	1	0.4 (0.01–2.5)	62	7.9 (6.2–10.0)	<0.001
20–29	168	16.6 (14.5–19.0)	28	12.4 (8.7–17.3)	140	17.8 (15.3–20.7)	
30–39	195	19.3 (17.0–21.8)	54	23.9 (18.8–29.9)	142	18.1 (15.6–20.9)	
40–49	153	15.1 (13.1–17.5)	43	19.0 (14.4–24.6)	110	14.0 (11.8–16.6)	
50–59	167	16.5 (14.4–18.9)	46	20.4 (15.6–26.1)	121	15.4 (13.1–18.1)	
60+	264	26.1 (23.5–28.9)	54	23.9 (18.8–29.9)	210	26.8 (23.8–30.0)	
**Marital status**							
Ever married	744	73.6 (70.8–76.2)	182	80.2 (74.5–84.8)	562	71.7 (68.4–74.7)	0.01
Never married	267	26.4 (23.8–29.2)	45	19.8 (15.2–25.5)	222	28.3 (25.3–31.6)	
**Occupational status**							
Active	627	62.0 (59.0–65.0)	144	63.7 (57.3–68.7)	483	61.5 (58.1–64.9)	0.6
Passive	384	38.0 (35.0–41.0)	82	36.3 (30.3–42.7)	302	38.5 (35.1–41.9)	
**Education level**							
Less than secondary	453	44.8 (41.8–47.9)	125	55.3 (48.8–61.7)	328	41.8 (38.4–45.3)	<0.001
Secondary or above	558	55.2 (52.1–58.2)	101	44.7 (38.4–51.2)	457	58.2 (54.7–61.6)	
**Income class**							
High	232	23.0 (20.5–25.6)	41	18.1 (13.7–23.7)	192	24.5 (21.6–27.6)	0.1
Medium	497	49.2 (46.1–52.2)	116	51.3 (44.8–57.8)	380	48.4 (44.9–51.9)	
Low	282	27.9 (25.2–30.7)	69	30.5 (24.9–36.8)	213	27.1 (24.1–30.4)	
**Number of household members**							
1	231	22.9 (20.4–25.5)	51	22.5 (17.5–28.3)	180	23.0 (20.2–26.0)	0.9
2 or more	780	77.1 (74.5–79.6)	176	77.5 (71.7–82.5)	604	77.0 (74.0–79.9)	
**Children in home**							
Yes	232	23.0 (20.5–25.6)	65	28.8 (23.3–35.0)	167	21.3 (18.6–24.3)	0.02
No	779	77.0 (74.4–79.5)	161	71.2 (65.0–76.8)	618	78.7 (75.7–81.5)	
**Place of residence**							
Rural	394	39.0 (36.0–42.0)	76	33.5 (27.7–39.9)	318	40.5 (37.1–44.0)	0.06
Urban	617	61.0 (58.0–64.0)	151	66.5 (60.2–72.3)	467	59.5 (56.0–62.9)	

**Table 2:** Proportion of homes with smoke-free rules and exposure to secondhand smoke (SHS) at home among in a representative sample aged 15 and over in Poland, by smoking status.

Type of Smoke-Free Rules	Smoke-Free Home Rules		*p*	Exposure to SHS at Home (Answered Yes)		*p*
	Smokers (n = 226)	Non-Smokers (n = 785)		Smokers (n = 226)	Non-Smokers (n = 785)	
	% (95% CI)	% (95% CI)		% (95% CI)	% (95% CI)	
Full rule	18.6 (14.1–24.2)	79.8 (76.8–82.4)	<0.001	2.4 (0.4–12.3)	1.8 (1.0–3.1)	<0.001
Partial rule	46.5 (40.1–53.0)	18.5 (15.9–21.3)		8.6 (4.6–15.5)	11.7 (7.5–18.0)	
No rules	35.0 (29.0–41.4)	1.8 (1.1–3.0)		20.3 (12.9–30.4)	57.1 (32.6–78.6)	

**Table 3:** Implementation of voluntary smoke-free home rules by selected socioeconomic factors in a representative sample aged 15 and over in Poland: Odds ratios (OR) and 95% confidence intervals (95% CI). (N = 1011).

Variable	Yes	Simple Regression	Multiple Regression		
			Model 1	Model 2	Model 3
	n (%)	OR (95% CI)	aOR (95% CI)		
**Smoking status**					
Smoker	42 (18.6)	Reference	Reference	Reference	Reference
Non-smoker	626 (79.8)	17.20 (11.79–25.07) **	18.81 (12.74–27.78) **	18.70 (12.62–27.72) **	19.17 (12.89–28.50) **
**Gender**					
Women	355 (67.4)	Reference	Reference	Reference	Reference
Men	313 (64.7)	0.89 (0.69–1.16)	1.11 (0.81–1.51)	1.11 (0.81–1.52)	1.14 (0.83–1.57)
**Age (years)**					
15–19	44 (69.8)	Reference	Reference	Reference	Reference
20–29	115 (68.5)	0.91 (0.49–1.71)	1.49 (0.77–2.90)	1.46 (0.73–2.93)	1.43 (0.71–2.88)
30–39	120 (61.2)	0.67 (0.36–1.24)	1.46 (0.76–2.81)	1.43 (0.72–2.83)	1.27 (0.62–2.57)
40–49	103 (67.3)	0.88 (0.47–1.67)	2.26 (1.12–4.56) *	2.21 (1.07–4.57) *	2.08 (0.99–4.32)
50–59	109 (64.9)	0.79 (0.42–1.47)	1.88 (0.95–3.72)	1.85 (0.92–3.72)	1.91 (0.94–3.88)
60+	177 (67.1)	0.87 (0.48–1.58)	1.62 (0.86–3.04)	1.60 (0.85–3.03)	1.63 (0.84–3.16)
**Education level**					
Less than secondary	282 (62.3)	Reference		Reference	Reference
Secondary or above	386 (69.2)	1.37 (1.05–1.78) *		1.04 (0.74–1.45)	1.04 (0.74–1.46)
**Number of household** **members**					
1	155 (67.1)	Reference			Reference
2 or more	514 (65.9)	0.95 (0.70–1.30)			0.91 (0.61–1.37)
**Children in home**					
No	518 (66.5)	Reference			Reference
Yes	150 (64.7)	0.93 (0.69–1.27)			1.36 (0.87–2.12)

* *p* < 0.05; ** *p* < 0.001.

**Table 4:** Exposure to secondhand smoke at home by selected socio-economic factors in a representative sample aged 15 and over in Poland: Odds ratios (OR) and 95% confidence intervals (95% CI). (N = 1011).

Variable	Yes	Simple Regression	Multiple Regression		
			Model 1	Model 2	Model 3
	n (%)	OR (95% CI)	aOR (95% CI)		
**Smoking status**					
Smoker	26 (11.5)	Reference	Reference	Reference	Reference
Non-smoker	36 (4.6)	0.37 (0.22–0.63) **	0.35 (0.20–0.60) **	0.36 (0.21–0.63) **	0.35 (0.20–0.61) **
**Gender**					
Women	31 (5.9)	Reference	Reference	Reference	Reference
Men	30 (6.2)	1.07 (0.64–1.79)	0.97 (0.58–1.64)	0.94 (0.56–1.59)	0.90 (0.53–1.53)
**Age (years)**					
15–29	16 (6.9)	Reference	Reference	Reference	Reference
30–49	20 (5.8)	0.82 (0.43–1.56)	0.70 (0.36–1.36)	0.65 (0.34–1.27)	0.64 (0.32–1.30)
50+	25 (5.8)	0.82 (0.42–1.60)	0.67 (0.33–1.33)	0.68 (0.34–1.37)	0.76 (0.37–1.56)
**Education level**					
Less than secondary	35 (7.7)	Reference		Reference	Reference
Secondary or above	27 (4.8)	0.62 (0.37–1.04)		0.66 (0.39–1.14)	0.66 (0.39–1.13)
**Number of household members**					
1	12 (5.2)	Reference			Reference
2 or more	50 (6.4)	1.26 (0.66–2.42)			1.33 (0.67–2.67)
**Children in home**					
No	50 (6.4)	Reference			Reference
Yes	12 (5.2)	0.80 (0.42–1.53)			0.64 (0.30–1.35)

** *p* < 0.001.
